# Tobacco Use Screening and Counseling During Hospital Outpatient Visits Among US Adults, 2005–2010

**DOI:** 10.5888/pcd12.140529

**Published:** 2015-08-20

**Authors:** Ahmed Jamal, Shanta R. Dube, Brian A. King

**Affiliations:** Author Affiliations: Shanta R. Dube, Brian A. King, Office on Smoking and Health, National Center for Chronic Disease Prevention and Health Promotion, Centers for Disease Control and Prevention, Atlanta, Georgia.

## Abstract

**Introduction:**

Physicians and health care providers play an important role in educating their patients about the health risks of tobacco use and in providing effective cessation interventions. Little is known about these practices in hospital outpatient settings. The objective of the study was to assess the prevalence, correlates, and trends of tobacco use screening and cessation assistance offered to US adults during their hospital outpatient clinic visits.

**Methods:**

Data for aggregated hospital outpatient visits among patients aged 18 years or older (N = 148,727) from the 2005–2010 National Hospital Ambulatory Medical Care Survey were analyzed. Tobacco use screening was defined as documentation of screening for either current tobacco use (cigarettes, cigars, snuff, or chewing tobacco) or no current use on the patient record form. Tobacco cessation assistance was defined as documentation of either tobacco counseling or cessation medications.

**Results:**

Tobacco use screening was reported for 63.0% (estimated 271 million visits) of hospital outpatient visits, and cessation assistance was reported for 24.5% (estimated 17.1 million visits) of visits among current tobacco users. From 2005 through 2010, tobacco use screening (*P* for trend = .06) and cessation assistance (*P* for trend = .17) did not change significantly.

**Conclusion:**

From 2005 through 2010, more than one-third of hospital outpatient visits had no screening for tobacco use, and among current tobacco users, only 1 in 4 received any cessation assistance. Health care providers should consistently identify and document their patients’ tobacco use status and provide them with appropriate tobacco cessation assistance. Opportunities also exist to expand the coverage for tobacco cessation.

## Introduction

Tobacco use is the leading cause of preventable disease and death in the United States. Cigarette smoking accounts for more than 480,000 premature deaths annually and costs the United States approximately $130 billion in direct medical expenses and $150 billion in lost productivity each year (1). Despite progress over the past several decades, declines in adult cigarette smoking have slowed in recent years (2,3). In 2012, current cigarette smoking among US adults was 18.1%, or nearly 42.1 million people (4). Moreover, the use of nontraditional tobacco products, including little cigars and electronic cigarettes, has become prevalent (5,6).

Health professionals play an important role in educating their patients about the health risks of tobacco use and in providing effective cessation interventions. Most smokers want to quit, health professionals have frequent contact and high credibility with smokers, and brief clinical interventions are effective in motivating and assisting tobacco users to quit (7). The 2008 update to the US Public Health Service Clinical Practice Guideline for Treating Tobacco Use and Dependence recommends that clinicians and health care delivery systems consistently identify and document tobacco use status and treat every tobacco user seen in a health care setting (7). Specifically, the guideline recommends the “5A” model to help patients quit using tobacco. The approach encourages health professionals to *ask* patients if they use tobacco, *advise* them to quit, *assess* their willingness to quit, *assist* them by providing or referring for counseling or additional treatment and by offering medication (unless contraindicated), and to *arrange* for follow-up contact to prevent relapse (7). However, effective screening and treatment of tobacco use are not consistently administered in clinical settings (7). Therefore, enhanced population-based and clinical efforts are warranted to prevent and reduce tobacco use in this setting (7,8).

The *Healthy People 2020* objectives for health systems change call for increasing tobacco screening in hospital ambulatory care settings to 66.2% (TU-9.2) and increasing tobacco cessation counseling in hospital ambulatory care settings to 24.9% (TU-10.2) (9). The National Quality Forum has also endorsed specific clinical quality measures on tobacco use assessment and cessation interventions (10).

Studies have assessed the extent of tobacco use screening and cessation assistance in physician outpatient settings (11–13); however, little is known about these practices in hospital outpatient settings, which account for approximately 1 in 10 outpatient visits. To address this gap, we analyzed aggregated data on hospital outpatient visits made by adults aged 18 years or older from the 2005–2010 National Hospital Ambulatory Medical Care Survey (NHAMCS) to determine the prevalence, correlates, and trends in screening for tobacco use and tobacco cessation assistance in the United States.

## Methods

We analyzed data from the 2005–2010 NHAMCS, the most recent aggregated public use data set available during this article preparation. The NHAMCS is a national probability sample of visits to the emergency and outpatient departments of noninstitutional general and short-stay hospitals (excluding federal, military, and Veterans Administration hospitals) in the 50 US states and the District of Columbia.

The basic sampling unit for NHAMCS is the patient visit or encounter, which is systematically selected over a randomly assigned 4-week reporting period. Although NHAMCS captures both emergency department (ED) and hospital outpatient visits, tobacco use status is captured only during hospital outpatient visits (ED visits do not include any information on patients’ tobacco use). As a result, the scope of our analyses was limited to hospital outpatient visits only. The 2005–2010 NHAMCS outpatient department (OPD) sample consisted of 148,727 hospital outpatient visits by people aged 18 years or older, ranging from 21,401 visits in 2005 to 26,345 in 2010. NHAMCS used a hierarchical scheme to determine the primary expected source of payment. During the 2005–2007 NHAMCS, respondents eligible for both Medicare and Medicaid were categorized as Medicaid recipients; however, these respondents were classified as Medicare recipients in the 2008–2010 NHAMCS. To account for this change, the 2005–2007 payment type was recoded to be consistent with the 2008–2010 classification for primary expected payment source (14).

Tobacco use was defined as smoking cigarettes or cigars, using snuff, or chewing tobacco. The status of a patient not currently using tobacco was marked “not current.” The status of a patient currently using tobacco was marked “current.” If it could not be determined whether the patient currently used or did not use tobacco, that patient’s status was marked “unknown.” Tobacco use screening during hospital outpatient visits was defined as documentation of screening for either current tobacco use or no current use on the patient record form (PRF). Tobacco cessation assistance was defined as documentation of either tobacco counseling or cessation medications during visits by persons aged 18 years or older who were current tobacco users. Tobacco counseling was defined as information given in the form of health education to the patient on topics related to tobacco use and exposure to secondhand smoke, information on smoking cessation and prevention of tobacco use, and referrals to other health professionals for smoking cessation programs. Provision of medications was identified through patient charts as medications that were ordered, supplied, administered, or continued. Data on medications were entered as free text for each visit, and medications were limited to no more than 8 prescription or over-the-counter tobacco cessation medications, which included nicotine replacement therapy (nicotine patch, gum, lozenge, nasal spray, or inhaler), bupropion, or varenicline. The hospital staff and census field representatives were responsible for completing PRFs for data abstraction.

Tobacco use screening and cessation assistance during visits were assessed by age (18–24 y, 25–44 y, 45–64 y, ≥65 y), sex, race/ethnicity (non-Hispanic white; non-Hispanic black; Hispanic; other race/multiple race, non-Hispanic), poverty level (percentage of people living at or below the federal level in a patient’s zip code), health insurance type (private insurance; Medicare, Medicaid or State Children’s Health Insurance Program [SCHIP]; no insurance; other), and tobacco use status (current tobacco user, nonuser). Assessed visit- and clinic-related characteristics included whether the physician/provider was the patient’s primary care physician (yes, no), major reason for visit (new problem <3-month onset; chronic problem; presurgery/postsurgery; preventive care), clinic type (general medicine, surgery, obstetrics and gynecology, substance abuse, other), and hospital electronic medical record use (yes, no).

Analyses were conducted using SAS version 9.2 (SAS Institute, Inc) and SUDAAN version 10.0.2 (RTI International) and restricted to records in which current tobacco use status was recorded. Data were adjusted for nonresponse and weighted to be nationally representative; 95% confidence intervals were calculated and estimates were considered significant if confidence intervals did not overlap. The overlapping confidence interval approach is not a formal statistical test for assessing differences; formal statistical testing may have resulted in different conclusions. Multivariate logistic regression with orthogonal polynomials was used to analyze linear trends from 2005 through 2010, adjusted for sex, age, and race/ethnicity (α = .05).

## Results

### Screening

**Figure 1:**
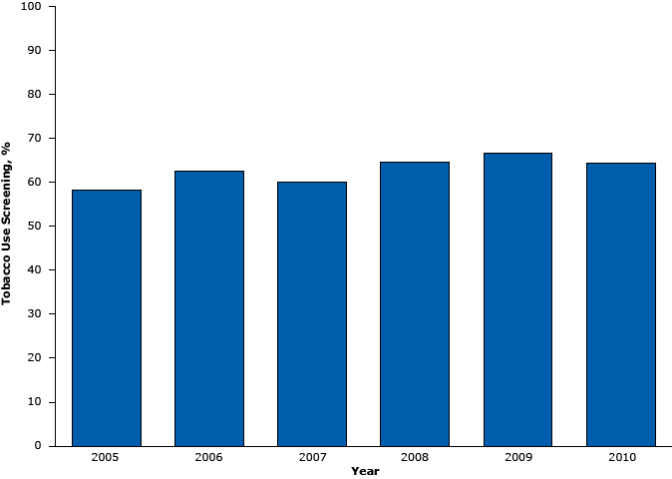
Percentage of tobacco use screening during hospital outpatient visits by adults aged 18 years or older, National Hospital Ambulatory Medical Care Survey, United States 2005–2010. **Table:** 

From 2005 through 2010, adults aged 18 years or older made, on average, 71.8 million hospital outpatient visits annually to hospital outpatient physicians (range, 62.9 million in 2005 to 80.3 million in 2008), or an estimated 431 million visits from 2005 through 2010 combined. On average, 45.2 million (63.0%) hospital outpatient visits included tobacco use screening each year, or an estimated 271 million visits from 2005 through 2010 combined (Table). Of the visits that included tobacco use screening, 25.7% (11.6 million annual average visits) were made by current tobacco users. From 2005 through 2010, tobacco use screening did not change over time after adjusting for sex, age, and race/ethnicity (*P* for trend = .06) (Figure 1). 

**Table:** Receipt of Tobacco Use Screening and Cessation Assistance During Hospital Outpatient Visits by Adults Aged ≥18 Years, by Patient and Hospital Characteristics, National Hospital Ambulatory Medical Care Survey, United States, 2005–2010

Characteristic	Tobacco Use Screening^a^ Recorded During Visit (n = 89,107)^b^	Visits by Current Tobacco Users^c^ (n = 23,061)	Visits by Current Tobacco Users With Tobacco Counseling,^d^ Cessation Medication,^e^ or Both (n = 5,451)
	% (95% Confidence Interval)		
**Total (n = 148,727)** f	63.0 (59.9–66.0)	25.7 (24.7–26.8)	24.5 (22.7–26.3)
**Patient age group, y**			
18–24	64.0 (60.2–67.7)	26.5 (23.5–29.4)	20.5 (16.6–24.5)
25–44	62.0 (58.9–65.1)	30.9 (28.8–33.0)	23.9 (20.9–26.8)
45–64	63.3 (60.2–66.4)	29.5 (28.0–31.0)	26.2 (23.6–28.8)
≥65	63.4 (59.5–67.2)	11.7 (10.7–12.7)	23.5 (20.1–26.9)
**Patient sex**			
Male	62.2 (58.9–65.5)	31.1 (29.2–33.0)	24.2 (21.3–27.1)
Female	63.4 (60.3–66.5)	22.8 (21.5–24.1)	24.6 (22.0–27.3)
**Patient race/ethnicity**			
White, non-Hispanic	65.1 (61.5–68.7)	26.1 (24.8–27.5)	23.3 (20.8–25.8)
Black, non-Hispanic	61.2 (57.4–65.1)	29.1 (26.8–31.4)	28.3 (24.4–32.1)
Hispanic	55.4 (50.9–59.9)	20.2 (17.4–22.9)	22.2 (18.1–26.2)
Other race/multiple race, non-Hispanic	64.2 (58.9–69.5)	19.1 (14.7–23.5)	28.5 (18.3–38.6)
**Patient health insurance^g^**			
Private insurance	65.3 (61.3–69.3)	21.1 (19.9–22.4)	21.8 (19.5–24.0)
Medicare	63.4 (59.8–67.0)	18.0 (16.8–19.1)	21.4 (18.7–24.0)
Medicaid/SCHIP	60.4 (56.4–64.4)	35.5 (32.1–38.9)	27.6 (24.7–30.4)
No insurance	66.7 (60.4–72.9)	34.9 (31.9–38.0)	29.3 (22.6–36.1)
Other	52.1 (47.9–56.3)	33.8 (30.9–36.8)	21.1 (16.7–25.5)
**Poverty % in patient's zip code^g^**			
<5.00	63.3 (58.6–68.1)	19.2 (17.3–21.2)	15.7 (13.0–18.3)
5.00–9.99	65.8 (61.3–70.2)	24.1 (22.3–26.0)	23.4 (20.5–26.3)
10.00–19.99	65.3 (61.1–69.6)	26.2 (24.6–27.8)	23.8 (20.9–26.8)
≥20.00	60.7 (56.7–64.6)	28.8 (26.3–31.2)	29.1 (24.8–33.3)
**Patient’s primary care physician/provider^g^**			
Yes	71.6 (67.3–75.8)	25.7 (24.1–27.3)	29.2 (25.4–33.1)
No	58.5 (55.4–61.6)	25.3 (23.3–27.2)	20.6 (17.8–23.4)
**Major reason for the visit^g^**			
New problem (<3-month onset)	67.9 (64.7–71.0)	28.4 (26.6–30.1)	20.7 (17.4–23.9)
Chronic problem	59.9 (56.4–63.4)	26.8 (24.9–28.7)	26.8 (24.3–29.4)
Presurgery or postsurgery	60.7 (54.8–66.7)	23.3 (20.7–25.8)	17.5 (12.5–22.5)
Preventive care	63.0 (58.2–67.8)	18.7 (16.9–20.5)	30.5 (27.1–33.9)
**Preventive care^g^**			
Yes	63.0 (58.2–67.8)	18.7 (16.9–20.5)	30.5 (27.1–33.9)
No	63.1 (60.0–66.2)	27.3 (25.8–28.7)	23.6 (21.1–26.0)
**Clinic type**			
General medicine	67.1 (64.1–70.1)	26.4 (24.8–27.9)	26.7 (23.4–29.9)
Surgery	55.7 (49.4–62.1)	23.0 (20.8–25.1)	12.7 (10.0–15.4)
Obstetrics and gynecology	62.8 (57.8–67.8)	18.7 (16.2–21.2)	24.3 (21.2–27.4)
Substance abuse	68.3 (56.9–79.8)	72.3 (68.7–75.9)	29.2 (12.7–45.7)
Other	45.2 (40.0–50.3)	29.2 (24.9–33.4)	19.5 (13.9–25.1)
**Hospital outpatient department uses electronic medical records^g^**			
Yes	61.5 (57.7–65.2)	25.4 (23.4–27.3)	22.6 (19.9–25.2)
No	61.8 (56.9–66.8)	28.8 (26.9–30.7)	27.7 (23.9–31.4)

Abbreviation: SCHIP, State Children’s Health Insurance Program.

a Visits during which current tobacco use (smoke cigarettes or cigars or use snuff or chewing tobacco) or no current use was recorded. Denominator includes current tobacco use, no current use, and unknown.

b Yearly visits with tobacco use screening: 12,773 in 2005; 14,484 in 2006; 14,640 in 2007; 15,923 in 2008; 14,839 in 2009; and 16,448 in 2010.

c Visits during which current tobacco use (smoke cigarettes or cigars or use snuff or chewing tobacco) was documented.

d Tobacco counseling refers to any information provided that related to tobacco use in any form, including cigarettes, cigars, snuff, and chewing tobacco, and on exposure to tobacco in the form of secondhand smoke, smoking cessation, and prevention of tobacco use, as well as referrals to other health care providers for smoking cessation programs.

e Cessation medications include nicotine replacement therapy (nicotine patch, gum, lozenge, nasal spray, and inhaler), bupropion, and varenicline.

f Yearly total visits: 21,401 in 2005; 24,743 in 2006; 26,062 in 2007; 25,261 in 2008; 24,915 in 2009; and 26,345 in 2010.

g Excludes unknown or blank entries for the covariate.

Year	Tobacco Use Screening, %
2005	58.3
2006	62.7
2007	60.2
2008	64.6
2009	66.8
2010	64.5

Tobacco use screening during hospital outpatient visits varied by patient’s race/ethnicity; visits made by Hispanics (55.4%) were less likely to receive tobacco use screening than those by non-Hispanic whites (65.1%). Patients who visited their primary care physician or provider were more likely to receive tobacco use screening (71.6%) than those who visited a physician who was not their primary care physician or provider (58.5%). Patients who made visits for a chronic problem (routine or flare-up) were less likely to receive tobacco use screening (59.9%) than those who visited with a new problem (<3 months onset) (67.9%). Patients who made visits to general medicine clinics (67.1%) were more likely to receive tobacco use screening than those who made visits to surgical clinics (55.7%) or clinics with other specialties (45.2%), excluding obstetrics and gynecology (62.8%) and substance abuse clinics (68.3%). Screening for tobacco use did not vary by poverty level.

### Current tobacco use

The proportion of visits made by adults who screened positive for current tobacco use varied by patient’s age, sex, and race/ethnicity. Current tobacco use was greater among those younger than 65 years (18–24 y, 26.5%; 25–44 y, 30.9%; 45–64 y, 29.5%) than those aged 65 years or older (11.7%), among men (31.1%) than women (22.8%), and among non-Hispanic whites (26.1%) and non-Hispanic blacks (29.1%) than Hispanics (20.2%). Patients who screened positive for current tobacco use also varied by health insurance type. Current tobacco use was greater among those with Medicaid or SCHIP benefits (35.5%) or no insurance (34.9%) than those with private insurance (21.1%) or Medicare (18.0%). Current tobacco use was higher among patients living in a high poverty zone (zip code with 5.00%–9.99% poverty, 24.1%; zip code with 10.00%–19.99% poverty, 26.2%; and zip code with ≥20.00% poverty, 28.8%) than those living in a low poverty zone (zip code with <5.00% poverty, 19.2%). Lower prevalence of tobacco use was observed among patients who made visits for preventive care (18.7%) than those who made visits for a new problem (28.4%), chronic problem (26.8%), presurgery/postsurgery (23.3%), or overall nonpreventive care (27.3%). Higher prevalence of tobacco use was also observed among those visiting substance abuse clinics (72.3%) than among those visiting all other types of clinics, including general medicine (26.4%), surgery (23.0%), obstetrics and gynecology (18.7%), and other clinics (29.2%). Current tobacco use decreased from 28.9% in 2005 to 22.6% in 2010 among hospital outpatient visits (*P *for trend <.001).

### Cessation assistance

**Figure 2:**
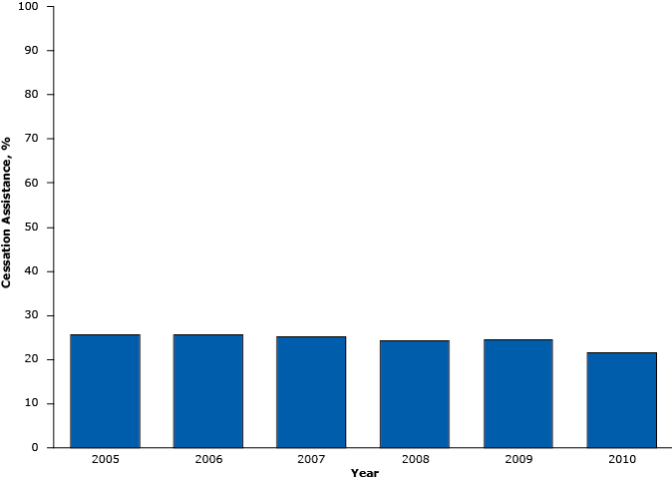
Percentage of cessation assistance (counseling, or medications, or both) ordered or provided during hospital outpatient visits by adults aged ≥18 years, National Hospital Ambulatory Medical Care Survey, United States 2005–2010. **Table:** 

Among patients who screened positive for current tobacco use, 24.5% (or an estimated 17.1 million visits) received any cessation assistance, including tobacco counseling, a prescription or order for a cessation medication at the visit, or both. Cessation assistance was higher for visits made by those with Medicaid/SCHIP (27.6%) than those with private insurance (21.8%) or Medicare (21.4%). Patients living in a high poverty zone were more likely to receive cessation assistance (zip code with 5.00%–9.99% poverty, 23.4% of visits; zip code with 10.00%–19.99% poverty, 23.8% of visits; and zip code with ≥20.00% poverty: 29.1% of visits) than those living in a low poverty zone (zip code with <5.00% poverty, 15.7% of visits). Receipt of cessation assistance was higher among those who visited their primary care physician (29.2% of visits) than those who visited a physician who was not their primary care physician (20.6% of visits). By major reason for the visit, cessation assistance was higher for preventive care (30.5% of visits) and chronic problems (26.8% of visits) than those for new problems (20.7% of visits), presurgery or postsurgery (17.5% of visits), and overall nonpreventive care (23.6% of visits). Higher prevalence of assistance was observed among patients who made a visit to general medicine clinics (26.7% of visits) than those made to surgical clinics (12.7% of visits). From 2005 through 2010, cessation assistance did not change over time after adjusting for sex, age, and race/ethnicity (*P* for trend = .17) (Figure 2).

Year	Cessation Assistance, %
2005	25.6
2006	25.6
2007	25.2
2008	24.3
2009	24.4
2010	21.6

## Discussion

We found that tobacco use screening occurred during most US adult visits to a hospital outpatient department from 2005 through 2010 (63.0%); however, screening did not occur in more than 1 in 3 visits. Moreover, among patients who were identified as current tobacco users, only 24.5% received any tobacco cessation assistance, including counseling, medications, or both. Consistent with the Public Health Service Clinical Practice Guideline, it is essential that clinicians and the health-care delivery systems consistently identify and document tobacco use status and treat every tobacco user with cessation counseling and US Food and Drug Administration (FDA)-approved tobacco cessation medications, except when medically contraindicated or with specific populations for which evidence of effectiveness of medications is insufficient, such as pregnant women, smokeless tobacco users, light smokers, and adolescents (7).

Disparities in tobacco use screening were observed across population groups. For example, tobacco use screening was lower among Hispanic patients than among non-Hispanic whites, a finding that is similar to a study of outpatient visits to office-based physicians from 2001 through 2005; in that study, lack of insurance or more new-patient visits did not explain this ethnic difference (15). The lower prevalence of tobacco use screening among Hispanic patients may be attributable to cultural and language differences between patients and physicians, which have previously been identified as barriers to cancer screening (16). To address these barriers to preventive services for Hispanic patients as well as other underrepresented patient populations, medical school curricula could include training programs for medical students in assisting patients whose primary language is not English (17,18). Although screening for tobacco use did not vary by poverty level, visits made by patients living in high poverty areas had higher rates of documented current tobacco use. This finding is consistent with population-based studies that document higher smoking prevalence among adults living below the federal poverty level (19). Furthermore, prevalence of current tobacco use was higher among visits made to substance abuse clinics than those made to other types of clinics, which is consistent with findings from existing research (20). Targeted efforts to enhance tobacco use screening and the provision of effective cessation treatments to people with substance use disorders can help reduce tobacco use and tobacco-related illness and death among this vulnerable population (8). Similarly, current tobacco use was lower for preventive care visits, which could be attributed to greater time being devoted to tobacco use screening and counseling during preventive care visits than nonpreventive care visits or to people with unhealthy lifestyles being less likely to seek preventive care.

Patients visiting their primary care physician or provider had a higher prevalence of receiving tobacco use screening and cessation counseling than patients who visited a physician who was not their primary care physician, perhaps because the primary care physicians were providing more routine care than specialized care, and tobacco cessation counseling may have been provided as part of a wellness or preventive care visit. Outpatient visits made by patients with a chronic problem were less likely to receive tobacco use screening than visits with a new problem (<3-month onset). This finding could be because patients having chronic conditions may experience more emphasis on receiving treatment by their physicians for their illness than on prevention (eg, tobacco use screening). From 2005 through 2010, hospital outpatient visits with no documented insurance or visits with Medicaid/SCHIP insurance had a higher prevalence of current tobacco use than those insured by private insurance or Medicare. However, neither private insurers nor state Medicaid programs consistently provide comprehensive coverage of evidence-based cessation treatments (8). For example, in 2014, although all 51 Medicaid programs covered some form of tobacco-dependence treatment for some Medicaid enrollees, only 7 states covered all 7 FDA-approved cessation medications and individual and group counseling for all Medicaid enrollees (21). Therefore, more efforts are warranted to meet the *Healthy People 2020* objective (TU-8) of increasing comprehensive Medicaid insurance coverage of evidence-based cessation treatments for nicotine dependency (9).

Ongoing changes in the US health care system offer opportunities to improve the use of clinical preventive services among adults. In particular, the Patient Protection and Affordable Care Act of 2010, as amended by the Health Care and Education Reconciliation Act of 2010 (referred to as the Affordable Care Act), and other national initiatives offer increasing tobacco use cessation treatment coverage (22). Effective January 2014, the Affordable Care Act prohibited traditional state Medicaid programs that cover prescription medications from excluding FDA-approved cessation medications, including over-the-counter medications, from Medicaid drug coverage. As of July 2011, Medicaid began allowing states to apply for 50% administrative match funds for telephone quitline services provided to Medicaid enrollees. Also as a part of the Affordable Care Act, as of January 2014, Medicaid expansion alternative benefit plans and newly qualified health insurance plans operating in the Health Insurance Marketplace are required to offer their members cessation coverage without cost sharing (23). The requirement to provide cessation coverage without cost sharing also applies to all private health plans, except for those that have continuously maintained “grandfathered” status since March 23, 2010 (24). The Marketplace allows eligible people and small businesses with up to 50 employees to purchase health insurance coverage from plans that meet criteria outlined in the Affordable Care Act. The federal government has established a Marketplace for states that have not yet created their own.

Several barriers can impede clinician assessment of and treatment for tobacco users, including lack of time, lack of knowledge of effective intervention strategies, inadequate payment for treatment, and lack of institutional support for routine assessment and treatment of tobacco use (3). Health care systems can support physician interventions by instituting effective systems-level changes that make screening for tobacco use and brief cessation interventions for tobacco users a standard part of every office visit. Provider-reminder systems can increase health care providers’ assessment and treatment of tobacco use in a range of clinical settings and populations. Provider reminder systems remind or prompt providers to screen and advise patients on tobacco use and cessation, and can be implemented as chart stickers, vital sign stamps, medical record flow sheets, check lists, or as part of electronic medical records (8,25).

This study is subject to at least 6 limitations. First, the definition of tobacco counseling included any information on tobacco use or secondhand smoke exposure, as well as referrals to tobacco cessation programs. Therefore, we could not assess the type of information provided or track the use of the 5A’s more precisely (3). Second, because bupropion can be prescribed as an antidepressant as well as for tobacco cessation, it is unclear which of these indications this medication was prescribed for. Third, tobacco counseling was only available for the current visit; therefore, it was not possible to determine the extent of health education during previous visits. Fourth, the study did not assess all types of tobacco use, including use of emerging products such as electronic cigarettes. Fifth, NHAMCS data were for 2005 through 2010; therefore, the estimates of tobacco use screening and cessation assistance may have changed. Finally, tobacco use screening may not have occurred at all visits by patients who had multiple visits to a sampled physician; thus, some visits with tobacco use screening or cessation assistance may have been missed.

Our study findings indicate that more than one-third (37.0%) of hospital outpatient visits, or 26.7 million annually, had no documentation of tobacco use status. Moreover, cessation assistance was not provided during 3 of 4 visits, or approximately 8.8 million annually. These findings underscore the need for enhanced efforts to encourage clinicians and the health care delivery systems to consistently identify and document tobacco use status and to treat every tobacco user with cessation counseling. The Centers for Disease Control and Prevention (CDC) recommends that states implement policies and other effective community-based strategies that increase tobacco cessation, in addition to working with health care systems, insurers, and purchasers of health insurance, to expand coverage for tobacco cessation and implement health system changes that support these effective clinical interventions (8,25). Other proven population-based interventions for increasing cessation include increasing the unit price of tobacco products, hard-hitting mass media campaigns, and comprehensive smoke-free policies in worksites and public places (8,25). Implementation of these proven policies and interventions, in concert with efforts to enhance tobacco use screening and the provision of cessation assistance in clinical settings, could result in a substantial reduction in tobacco use and tobacco-related illness and death in the United States (8).
