# Erratum, Vol. 12, August 20 Release

**DOI:** 10.5888/pcd12.140529e

**Published:** 2015-11-25

In the article “Tobacco Use Screening and Counseling During Hospital Outpatient Visits Among US Adults, 2005–2010,” the caption for Figure 2 was incorrectly phrased by the author. The original caption read as follows: “Percentage of cessation assistance (counseling, or medications, or both) ordered or provided during hospital outpatient visits by adults aged ≥18 years, National Hospital Ambulatory Medical Care Survey, United States 2005–2010.” The correct caption is as follows: “Percentage of cessation assistance (counseling, or medications, or both) ordered or provided during hospital outpatient visits by current tobacco users aged ≥18 years, National Hospital Ambulatory Medical Care Survey, United States 2005–2010.” The change was made to our website on November 10, 2015, and can be accessed at http://www.cdc.gov/pcd/issues/2015/14_0529.htm. We regret any confusion this error may have caused.

